# A Cross-Sectional Study of Dietary and Genetic Predictors of Blood Folate Levels in Healthy Young Adults

**DOI:** 10.3390/nu9090994

**Published:** 2017-09-08

**Authors:** Daniel Cummings, Kevin F. Dowling, Noah J. Silverstein, Alexandra S. Tanner, Hamdi Eryilmaz, Jordan W. Smoller, Joshua L. Roffman

**Affiliations:** 1 Department of Biopsychology, Tufts University, 419 Boston Ave, Medford, MA 02155, USA; daniel.cummings1@gmail.com; 2 Department of Psychiatry, Massachusetts General Hospital and Harvard Medical School, 149 13th Street, Charlestown, MA 02129, USA; kfdowling@mgh.harvard.edu (K.F.D.); noah.silverstein@umassmed.edu (N.J.S.); alexandra.s.tanner@gmail.com (A.S.T.); hamdi.eryilmaz@mgh.harvard.edu (H.E.); jsmoller@partners.org (J.W.S.)

**Keywords:** *FOLH1*, dietary folate, RBC folate, folic acid

## Abstract

Since 1998, the U.S. has mandated folic acid (FA) fortification of certain grain products to reduce the risk of neural tube defects. Folate intake and red blood cell (RBC) folate concentrations increased substantially post-intervention, although recent studies raise concerns about the level of ongoing benefit. This study investigated blood folate level determinants in healthy young adults, including intake of naturally occurring food folate, synthetic FA, and the interaction of naturally occurring food folate with a common missense variant in the *FOLH1* gene thought to affect absorption. Participants (*n* = 265) completed the Diet History Questionnaire II, RBC folate testing, and were genotyped for the 484T>C *FOLH1* variant. Men reported significantly greater intake of all folate sources except for supplemental FA, but RBC folate levels did not significantly differ by sex. Synthetic FA was a stronger predictor of RBC folate than naturally occurring food folate. In the largest racial group, synthetic FA and the interaction of *FOLH1* genotype with naturally occurring food folate significantly predicted RBC folate, with the overall model accounting for 13.8% of the variance in RBC folate levels. Blood folate levels rely on a complex interaction of natural and synthetic folate intake as well as *FOLH1* genotype.

## 1. Introduction

Folate refers to a diverse set of water-soluble vitamers with different oxidation states, one-carbon substitutions, and numbers of conjugated glutamates [1,2] (Table S1). Dietary sources of folate include leafy green vegetables, legumes, and fortified grain products. Folate bioavailability depends not only on dietary intake but also on cellular mechanisms mediating absorption and metabolism, as well as genetic polymorphisms of key enzymes or carriers in the folate pathway [3]. For example, folate hydrolase (*FOLH1*, also known as glutamate carboxypeptidase II, GCP-II) is an enzyme located on the intestinal brush border that must cleave the polyglutamated tail from naturally occurring food folate before it can be absorbed [4,5].

Folate is critical for a variety of cellular pathways [2], including the production of nucleotides and neurotransmitters [1], histone methylation and thus regulation of gene expression [6], and the methylation of homocysteine to methionine [7]. Given that folate requirements are higher during times of increased cell division to accommodate key cellular processes, it follows that folate is vital during pregnancy [8]. Perhaps the most well studied benefit of folate is its reduction of risk for neural tube defects (NTDs), which can occur in developing embryos before the neural tube closes between 23 and 27 days after conception [2,7]. Furthermore, reduced periconceptional folic acid intake has also been associated with higher risk for other conditions, such as severe language delay and autism [9,10].

Following evidence that supplemental folic acid reduces the risk of NTD-affected pregnancy [11,12], in 1992 the U.S. Public Health Service advised all women of child-bearing age to consume at least 400 mcg of folic acid each day [13]. Subsequently, the U.S. Food and Drug Administration mandated the fortification of enriched grain products with 140 mcg of folic acid per 100 g of cereal product beginning in January of 1998. Similar interventions have since been widely implemented in other Western hemisphere countries [14]. Fortified grain products and supplements (vitamins) contain synthetic folic acid, which has a higher bioavailability than naturally occurring food folate since it does not contain a polyglutamated tail [4]. To accommodate for the fact that synthetic folic acid is 1.7 times more bioavailable than naturally occurring food folate [15,16], folate levels are expressed as dietary folate equivalents (DFE), where 1 mcg of food folate equates to 1 mcg DFE, but 1 mcg of synthetic folic acid is 1.7 mcg DFE [17].

While the FDA predicted that mandatory grain fortification would increase folic acid consumption by about 100 mcg DFE per day [18,19], actual folic acid intake from fortified foods among non-supplement users increased by nearly 200 mcg DFE per day within a year after fortification was implemented [20]. Consequently the percentage of individuals with folate intake below the Estimated Average Requirement (EAR; defined as the daily intake of a nutrient adequate to meet the requirement of 50 percent of the population) of 320 mcg DFE/day decreased from 49 percent to 7 percent [20].

Blood folate levels can readily be assessed using serum or red blood cell (RBC) folate concentrations. Serum levels are responsive to immediate folate intake, whereas the RBC concentration reflects long-term folate status and is used by the Institute of Medicine as the primary indicator of folate adequacy [17]. Serum and RBC concentrations were, respectively, 2.5 and 1.5 times higher post-fortification (1999–2010) than pre-fortification (1988–1994), and the rates of folate deficiency (<10 nmol/L and <340 nmol/L for serum and RBC folate, respectively) fell to below 1 percent [21]. Furthermore, men and women have been found to differ in terms of intake and subsequent blood folate concentrations. While men have generally been shown to have higher dietary and total folate intakes than women [22,23,24,25], several studies have shown that baseline serum and RBC folate levels are the same or higher in women [19,21,23,24,25]. Additionally, two randomized placebo-controlled trials showed that women had larger increases in RBC folate concentrations than men when given the same doses of supplemental folic acid, suggesting men need to consume more folic acid than women to achieve the same RBC folate concentration [23,24,25]. In addition to sex, several single nucleotide polymorphisms (SNPs) in genes related to folate metabolism have been associated with variance in serum and RBC folate concentrations. For example, the 484T>C variant (rs202676) of *FOLH1*, a common missense polymorphism located within exon 2 of the gene, has been associated with lower RBC folate levels despite equivalent folate intake across genotypic groups [26].

Although the rollout of folic acid fortification was initially associated with both increased blood folate levels and a reduction of between 19 and 50 percent in NTDs [27,28], subsequent research has raised concerns about the level of ongoing benefit. Specifically, data from the National Health and Nutrition Examination Survey (NHANES) indicate that median serum and RBC folate concentrations among non-pregnant women of childbearing age decreased by 16 and 8 percent, respectively, between 1999–2000 and 2003–2004 [29]. Similar decreases (<10%) were observed between 1999–2002 and 2003–2006 [30]. Most recently, both serum and RBC folate concentrations decreased by 17 and 12 percent, respectively, between their initial peaks in 1999–2000 and 2009–2010 [21]. Such trends are reflected by changes in prevalence estimates of elevated plasma homocysteine levels, which significantly increased from 4.8% immediately following fortification (1999–2000) to 8.6% later in the post-fortification era (2007–2010); critically, over this same period, prevalence estimates of folate insufficiency based on elevated risk for NTD-affected pregnancy increased significantly from 15 to 23% [31,32]. While the prevalence of clinical folate deficiency—based on hematologic indicators of megaloblastic anemia—which is considered the final stage of folate deficiency [33] remained <1% during this period, the marked increase in folate insufficiency among women of reproductive age is of concern, as maternal folate levels necessary to prevent NTDs may be substantially higher than the cutoff for clinical folate deficiency.

Though the cause for this decrease in RBC folate levels remains unclear, some have suggested this drop reflects a reduction in the amount of folic acid added to fortified grain products [34]. Specifically, fortified grain products collected and analyzed immediately following fortification contained significantly greater total folic acid than mandated by federal regulations [35]. Despite this initial overage, subsequent studies suggest the folic acid content in fortified bread decreased between 2001 and 2003 [36], while other fortified foods may have folic acid content below federal regulations [37]. Though the use of different assays prevents direct comparison of these findings, it is possible that a decrease in RBC folate levels over time reflects underlying changes in the quantity of folic acid added to foods through fortification.

It is currently unclear whether the population is consuming sufficient folate to meet the EAR of 320 mcg DFE, 330 mcg (DFE) for those under 19 [17]. One particularly concerning finding is that, even when all sources of folate intake were considered, a sizeable percentage of women of child-bearing age were below the EAR, with 19 percent of 14–18 year olds below 330 mcg (DFE), with 17 percent of 19–30 year olds, and 15 percent of 31–50 year olds below 320 mcg (DFE) per day [22]. Furthermore, a recent review of 2007–2010 NHANES data found fortified grain products were the sole source of folic acid for 48.4% of women of childbearing age, 32% of whom were considered to be at elevated or high risk for NTD-affected pregnancy [32]. Given the variable contributions of different dietary sources of folate and folic acid to RBC folate, it is important to monitor changes in the sources of folate intake (naturally occurring food folate, folic acid from fortified foods, or supplemental folic acid) to ensure population dietary needs are met. Such monitoring is especially important given recent evidence showing that without folic acid only 12% of the population had usual intake above the EAR [18]. Additionally, as the relationship between dietary folate forms and blood folate status remains incompletely characterized, it is important to clarify the contributions made by different folate forms to RBC folate levels and to assess the effects of other factors—including common genetic variants in the folate pathway—that may influence this relationship.

As recent evidence suggests that population blood folate levels may be declining over time, it is increasingly important to identify factors that may interact with folate intake to affect the risk of NTD-affected births or subsequent risk for other neurodevelopmental disorders [9,10]. Though larger cohorts have previously examined the interplay of natural and synthetic folate forms on blood folate level, to our knowledge this is the first study to examine the combined influence of the *FOLH1* 484T>C polymorphism and dietary folate forms on RBC folate status. We predicted that blood folate level would be more strongly dependent on synthetic folic acid intake (both folic acid from fortified foods and supplemental folic acid) than on naturally occurring food folate intake, given that genetic variants in *FOLH1* across the population might stratify the efficiency of naturally occurring food folate absorption. We also specifically examined the interaction between the 484T>C variant in *FOLH1* and naturally occurring food folate intake. As *FOLH1* is critical for cleaving the polyglutamate tails from naturally occurring food folate we predicted there would be a stronger positive relationship between naturally occurring food folate intake and RBC folate levels for homozygous T-allele individuals than for those carrying the hypofunctional C-allele.

## 2. Materials and Methods

### 2.1. Participants

The 265 study participants (118 males) were healthy young adults, aged 17–34 years (mean = 23.6, standard deviation = 4.4). Participants were recruited from the Boston, MA metro area through Craigslist (60.0%), Facebook advertisements (12.5%), and hospital-wide electronic research recruitment resources (27.5%; Table S2) as part of a study of nutrition and MRI measures (MRI results to be reported separately). Research coordinators conducted a phone screening before scheduling participants for an appointment. Exclusionary criteria included current or past Axis I disorder (evaluated using the Structured Clinical Interview for DSM-IV Axis I Disorders-Non Patient Edition for adults and an abbreviated version of the Kiddie-SADS-Present and Lifetime Version for 17 to 18-years-old), contraindication to an MRI, current use of street drugs, consumption of >2 alcoholic beverages per day or >10 per week, use of psychotropic medications, or any medical or neurological condition that in the opinion of the principal investigator would potentially influence MRI results or folate metabolism through medication use (e.g., anticonvulsants, antihypertensives, methotrexate). All participants were fluent in English. The study was conducted in accordance with the Declaration of Helsinki, and study procedures were approved by the Partners HealthCare Human Subjects Committee (2013P001258) and all adult participants provided written informed consent. For minor subjects (age 17), the participant’s parent or legal guardian provided written informed consent, and the participant provided written assent.

### 2.2. Study Procedures

Intake of food and dietary supplements over the preceding year was surveyed using a web-based version of the National Cancer Institute Diet History Questionnaire II (DHQ) (http://appliedresearch.cancer.gov/dhq2/). Daily nutrient intake, including intake of folate from naturally occurring food folate or synthetic sources (folic acid from fortified foods or supplemental folic acid) was then calculated using an analysis software (Diet*Calc version 1.5.0, National Cancer Institute, Bethesda, MD, USA). This analysis software generates quantitative nutrient and food group intake estimates by referencing reported intake with several nutrient and food group databases including the USDA Food and Nutrient Database for Dietary Studies, the USDA MyPyramid Equivalents Database, and the Nutrition Data System for Research [38]. A trained phlebotomist drew non-fasting whole blood for RBC folate measurements. Per Institute of Medicine recommendations, RBC folate, rather than serum folate, was chosen as the primary outcome measure as it may be a better index of long-term folate status and folate adequacy [17]. RBC folate analysis was conducted with an electrochemiluminescence immunoassay (ECLIA; Elecsys Folate RBC binding assay, Roche Diagnostics, Indianapolis; mean coefficient of variation = 5.0%). DNA was extracted from blood or saliva samples at the Massachusetts General Hospital Psychiatric and Neurodevelopmental Genetics Unit and genotyped for the 484 T>C *FOLH1* variant (rs202676) using the Sequenom MassArray platform as previously described [3,26].

### 2.3. Statistical Analysis

Statistical analyses were completed using STATA version 14.1 (StataCorp, 2015, College Station, TX, USA) and R version 3.1.1 (R Foundation for Statistical Computing, 2014, Vienna, Austria). The R program ‘psych,’ version 1.5.8, was used to supplement correlation analyses [39]. Folate intake values are reported in mcg DFE, enabling direct comparison of food and synthetic folate intake. Comparisons between male and female subjects for demographics, genotype, and folate measures were conducted using unpaired *t*-tests or chi-square as appropriate. Folate intake measures that were not normally distributed were log-transformed for subsequent analysis. For non-normal folate intake measures that could not be transformed (i.e., consumption of 0 mcg DFE supplemental folic acid) a Mann–Whitney–Wilcoxon test was used. Synthetic folic acid intake (i.e., folic acid from fortified foods or supplemental folic acid) was merged into a single variable to facilitate comparison with natural folate intake. Hierarchical linear regression with R^2^ change was used to assess the dependence of RBC folate level on naturally occurring food folate intake, followed by sequential entry of synthetic folic acid intake, *FOLH1* genotype (T/T versus C-carrier), and genotype x naturally occurring food folate intake interaction as additional predictors. We repeated regression analyses using only subjects in the largest racial group (Caucasians) to control for potential confounding effects of population stratification artifact on genotype findings [40,41,42,43]. Given the dependence of folate intake on sex (described below), hierarchical regressions were then repeated using male and female participants separately. As *MTHFR* 677C>T polymorphisms may influence the measurement of RBC folate for some assays [44,45,46], additional analyses were conducted (as described in the Supplement) to determine if the results may be influenced by *MTHFR* genotype. For all analyses, the *p*-values reported are two-tailed. The datasets used and/or analyzed during the current study are available from the corresponding author on reasonable request.

## 3. Results

### 3.1. Demographics

Table 1 shows basic demographic information for the total sample, as well as separately for male and female participants. The majority of participants self-identified as White/Caucasian (*n* = 176) and non-Hispanic (*n* = 237), while Asian Americans, Black/African Americans, and other racial groups were represented by 42, 23, and 24 participants, respectively. Nearly all participants (97.7%) were non-smokers.

### 3.2. Folate Intake

Of participants who completed the dietary questionnaire (*n* = 264), the total mean daily folate intake from all sources across the study sample was 797 mcg DFE (*SD* = 465), comprising 332 mcg DFE (*SD* = 206) from naturally occurring food folate, 340 mcg DFE (*SD* = 245) from folic acid from fortified foods, and 125 mcg DFE (*SD* = 236) from supplemental folic acid. We then tested to see how many individuals were below the EAR of 320 mcg DFE, or 330 mcg DFE if under the age of 19 [17]. Of 264 individuals, 157 (59.4%) would have been under the EAR if only naturally occurring food folate intake were considered, 37 individuals (14.0%) would have been below the EAR if food folate intake (where food folate represents both naturally occurring food folate and folic acid from fortified foods) was included. Finally, when considering intake of folate from all sources, 27 individuals (10.2%) were under the EAR. One participant did not complete the entirety of the nutrition questionnaire and was excluded from these calculations.

Men and women differed significantly in folate intake (see Table 2). When compared to women, men had significantly greater total folate from all sources, food folate, and fortified folic acid intake. However, there was no significant difference in supplemental folic acid intake between sexes.

We then tested to see if there were any differences in the percentage of men and women who were below the EAR based on naturally occurring food folate alone, naturally occurring food folate and folic acid from fortified foods (food folate), and folate intake from all sources (see Table 3). Though a greater percentage of women than men failed to achieve the EAR when considering only naturally occurring food folate, the difference was not significant. However, based on intake of food folate, a greater percentage of women than men failed to reach the EAR (*p* = 0.0015). When considering folate intake from all sources, the percentage of women below EAR remained higher than that of men, a difference that approached significance (*p* = 0.068). However, as described in the discussion, these values are only approximations and thus considerable caution must be taken when interpreting these results relative to the EAR.

### 3.3. RBC Folate Concentration

Average RBC folate concentration across all participants with valid RBC folate results (*n* = 188) was 976.4 ng/mL (*SD* = 139.5). Seventy-six participants were excluded from this analysis due to unsuccessful phlebotomy attempts, low blood-volume specimens, improper transport of frozen specimens by lab couriers, specimen hemolysis, and due to specimens that were lost or improperly stored by the laboratory. Despite having greater folate intake, male RBC folate levels (*M* = 972.3 ng/mL, *SD* = 129.5) did not significantly differ from female RBC folate levels (*M* = 979.9 ng/mL, *SD* = 148.2), *t*(186) = 0.37, *p* = 0.71.

### 3.4. Predictors of RBC Folate Concentration

To assess the influence of genotype and folate intake on RBC concentration, we included only participants who were successfully genotyped and had valid RBC measurements in this analysis (*n* = 180; one participant failed to complete the dietary questionnaire, seven participants were not genotyped due to low sample volume, sample leakage, or missed collection). Participants included in the regression did not significantly differ from those who were excluded due to missing data with respect to age, sex, education, *FOLH1* genotype, and proportion of Caucasians (Table S3). Table 4 shows the hierarchical linear regression created to estimate RBC folate concentration, entering first naturally occurring food folate intake only, then adding total synthetic folic acid intake (from supplemental folic acid and folic acid from fortified foods), genotype, and the genotype x naturally occurring food folate intake interaction term sequentially.

By itself, naturally occurring food folate (Model 1) did not significantly predict RBC folate concentration (*p* = 0.37). When total synthetic folic acid intake was added as a predictor (Model 2), synthetic but not naturally occurring food folate intake significantly predicted RBC folate concentration, and the overall model explained significantly greater variance in RBC folate levels (R^2^ change = 0.074, *p* < 0.001). The addition of genotype as a predictor of RBC folate strengthened the model such that the change variance explained by Model 3 approached significance (R^2^ change = 0.011, *p* = 0.078). Finally, when the genotype x naturally occurring food folate intake interaction term was added (Model 4) it did not significantly predict RBC folate levels, nor was there any significant improvement in the variance explained by the whole model. With all predictors included, the model was significant (*p* < 0.001) and accounted for 8% of the variance in RBC folate levels.

Synthetic folic acid should not rely on *FOLH1* for intestinal absorption; accordingly, it was hypothesized that *FOLH1* genotype would not interact with total synthetic folic acid intake to influence RBC folate level. To test this hypothesis, an additional model was considered that added a genotype x total synthetic folic acid intake term. As expected, the model did not account for significantly more variance than Model 4 above (R^2^ change = −0.005, *p* = 0.88) and the genotype x total synthetic folic acid interaction was not significant (*t* = −0.15, *p* = 0.88).

In post-hoc analyses, we further separated total synthetic folic acid into folic acid from fortified foods and supplemental folic acid and included the log of each variable in separate regressions with RBC folate. As a number of participants did not take vitamin supplements, supplemental folic acid intake data could not be log transformed directly. Consequently, a constant of one was added to each supplemental folic acid value prior to transformation. Both the logs of supplemental folic acid intake and folic acid from fortified foods significantly predicted RBC folate (see Table S4).

**Figure 1:**
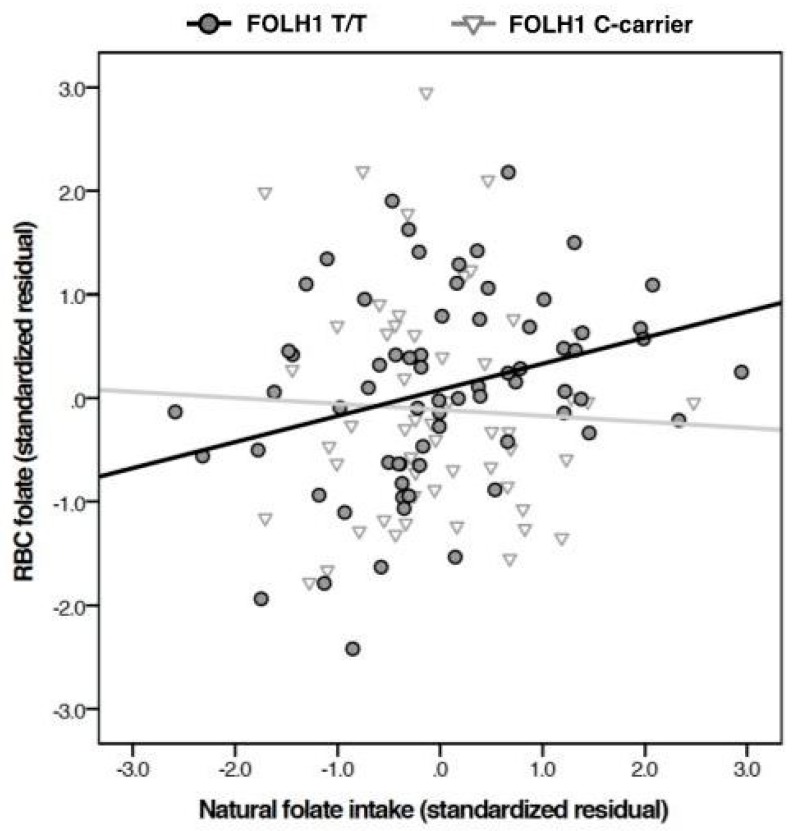
Interactive effect of food folate intake and *FOLH1* genotype on blood RBC folate levels for Caucasian participants. Naturally occurring food folate intake correlated positively with RBC folate level only among individuals homozygous for the 484T allele (interaction *p* = 0.043).

To control for potential confounding effects of population stratification on genotype findings [47], the original four-model hierarchical regression was repeated using only the subjects in the largest self-reported racial group (Caucasian, *n* = 123). The results of this analysis (see Table 5) deviated from those of the un-stratified model (see Table 4). Specifically, the inclusion of the genotype x naturally occurring food folate intake term significantly predicted both RBC folate and significantly strengthened the overall model (R^2^ change = 0.023, *p* = 0.043). The final model was significant and accounted for 13.8% of the variance in RBC folate levels (*p* < 0.001). Otherwise, the Caucasian-stratified results followed a similar pattern to the non-stratified sample (R^2^ change from Model 1 to Model 2 = 0.121, *p* < 0.001). A plot of the interaction (Figure 1) indicates that naturally occurring food folate intake is positively related to RBC folate levels only among individuals homozygous for the 484T allele. Again, a fifth model was constructed to test the interaction between *FOLH1* genotype and total synthetic folic acid intake. As hypothesized, the model did not account for significantly more variance (R^2^ change = 0.009, *p* = 0.29) and the interaction remained non-significant (*t* = −1.06, *p* = 0.29).

Finally, we repeated the four-model hierarchical regression separately for Caucasian males (*n* = 65) and females (*n* = 58). For males, only the addition of total synthetic folic acid intake significantly strengthened the overall model (Model 1 to Model 2 R^2^ change = 0.005, *p* = 0.02) and the final model explained 7.8% of the variance in RBC folate levels (Model 4 adjusted R^2^ = 0.078). This pattern of results was largely similar for female participants; however, unlike males, the interaction of *FOLH1* with naturally occurring food folate intake significantly strengthened the overall model for females, which explained 29% of the variance in RBC folate levels (see Table 6). To test whether this represented a significant difference an additional model was constructed (building on that described in Table 5), which included a three-way interaction of sex, *FOLH1* genotype, and the log of naturally occurring food folate. This model did not explain significantly more variance in RBC folate (R^2^ change = −0.0029, *p* = 0.29) and the interaction term was non-significant (*t* = −0.78, *p* = 0.44).

## 4. Discussion

Understanding why circulating folate levels vary from person to person is critical to ensuring adequate bioavailability, especially among women of childbearing age. This cross-sectional study of folate intake patterns in healthy young adults demonstrates the importance of separately considering the contributions of naturally occurring food folate and synthetic folic acid intake to circulating folate levels, especially when viewed in concert with sex and genetic variation in folate absorption.

On average, study participants reported receiving approximately equal amounts naturally occurring food folate (332 mcg) and folic acid from fortified foods (340 mcg DFE). Furthermore, 60.8% participants did not take supplemental folic acid (56.5% of all women, 66.1% of all men), while the 39.2% who did reported an average supplemental folic acid intake of 322 mcg DFE. Synthetic folic acid was critical to maintaining recommended folate intake, as 58.7% of participants (63.3% of female participants) would have been below the EAR of 320 mcg (330 for those under 19) based solely on naturally occurring food folate intake. This supports other recent evidence of the continued importance of fortification, with reports that the percentage of the population below the EAR fell from 88% with naturally occurring food folate alone to 11% with the addition of fortified folic acid [18]. In the present study, although fortification helped a large proportion of participants to attain sufficient folate intake, 10.2% of all individuals (13.6% of female participants) remained under the EAR even when considering folate intake from all sources. However, the percentage of participants above and below the EAR should be interpreted as approximates [48]. Thus, the present results are best understood as providing support for previous research that reiterated the importance of folic acid fortification to ensuring adequate folate levels for NTD prevention [22].

Consistent with previous reports [22,23,24,25], male and female participants differed substantially in folate intake, but did not differ in RBC folate levels. Men had significantly greater folate intake than women from all sources except supplements, for which intake did not differ by sex. Additionally, the frequency of inadequate folate intake did not differ significantly between women and men when considering only naturally occurring food folate intake. However, the proportion of women under the EAR was significantly greater when accounting for folic acid intake from fortified foods, and approached significance when considering intake of folate from all sources. It remains uncertain why women demonstrated equivalent RBC folate levels to men despite lower folate intake, although other authors have postulated that this pattern reflects increased average volume of distribution in men or potential differences in lean body mass [25].

In the second part of our analysis, we examined the relative contributions of naturally occurring food folate, synthetic folate, and a common genetic variant in *FOLH1* to circulating folate levels. Compared to serum folate, RBC folate is thought to provide a better estimate of folate levels within body tissues [49], where it plays a critical role in methylation reactions that regulate gene expression [6], homocysteine metabolism [50], neurotransmitter synthesis and turnover [1], and a host of other essential processes. However, relative to serum folate, RBC folate measurement may be considerably more vulnerable to small variations in sample storage and analysis, which may make it a less reliable measure of folate status [46,51].

As seen in Table 4, total synthetic folic acid intake is a stronger predictor of RBC folate level than is intake of naturally occurring food folate. Given that greater RBC folate levels reduce the risk of NTD-affected births [31,32], it is possible that greater intake of synthetic folic acid forms afford greater protection against NTDs (and potentially lower the risk for certain conditions such as autism and severe language delay [9,10]). Furthermore this difference between synthetic folic acid and naturally occurring food folate forms likely reflects the fact that, unlike synthetic folic acid, naturally occurring food folate must undergo hydrolysis of a polyglutamated tail moiety by *FOLH1*, a glutamate carboxypeptidase that lies within the intestinal brush border, before absorption. The 484T>C variant codes for a missense mutation (75Tyr>His) in the *FOLH1* enzyme. The functionality of this variant has not been established using biochemical assays, although structural modeling suggests that the C allele reduces folate binding [52]. Further, the C allele has previously been associated with reduced blood folate levels [26], suggesting that it is a hypofunctional variant that limits naturally occurring food folate absorption. As expected, *FOLH1* variants did not influence absorption of synthetic folic acid as such forms lack a polyglutamate tail that must be enzymatically cleaved by *FOLH1* prior to absorption.

In the present sample, which is smaller than those previously examined, the 484T>C genotype by itself did not contribute to RBC folate level. However, in the largest racial group, the interactive effect of genotype with naturally occurring food folate intake on RBC folate levels occurred in the anticipated direction. Specifically, the correlation between naturally occurring food folate and RBC folate was stronger among individuals homozygous for the T allele. This pattern suggests that polyglutamated folate (naturally occurring food folate) is less well absorbed among C allele carriers. This interpretation is consistent with results from previous research, which found those with hypofunctional *FOLH1* 484 variants had lower RBC folate levels despite equivalent dietary folate intake [26].

We repeated our analysis in the largest racial group so as to control for possible population stratification effects [47], a type of confounding wherein allele frequency varies across subgroups within a population in a manner that might confound the relationship between a genetic variant and a relevant phenotype. Pertinent to this study, the frequency of folate-related alleles has been shown to vary considerably amongst different racial subgroups [40,41,42,43], which could potentially confound *FOLH1* findings. As such, consistent with prior recommendations [47], we repeated our analyses in the largest mono-racial group so as to account for differences in frequency of folate-related SNPs amongst different genetic ancestries that could affect folate status [40,41,42,43,47].

Though the lack of a main effect of *FOLH1* genotype on RBC folate status may seem surprising, prior research has demonstrated that the effects of certain folate-related SNPs may be attenuated in high-folate intake samples [53]. As most participants reported considerable intake of synthetic folic acid, which bypasses the *FOLH1* carboxypeptidase and is directly absorbed into the body, the extent to which the *FOLH1* 484 T>C polymorphism may influence folate status in this population is likely a function of both the polymorphism but also on the amount of naturally occurring food folate intake in each participant’s diet. For instance, a participant who consumed more naturally occurring food folate but had the hypofunctional *FOLH1* variant might absorb less than participants with the functional *FOLH1* variant. However, this polymorphism would not have as great an effect on blood folate levels for those who derive most of their daily folate from synthetic sources, for which absorption is not *FOLH1* dependent.

After conducting the regression analysis separately by sex, it appears that overall relationships between intake (from either naturally occurring food folate or synthetic folic acid) and genotype may have been driven by the female subsample. The results of our hierarchical linear regression analyses followed the same pattern in both the full sample and the female subsample, with total synthetic folic acid intake and the genotype by naturally occurring food folate interaction significantly predicting RBC folate concentration. However, of the four models, only the inclusion of total synthetic folic acid significantly predicted RBC folate concentrations in males. If significant gender effects are found in subsequent studies of this polymorphism these results could reflect sex differences in folate metabolism, folate absorption, *FOLH1* expression, or, as previously reported, lean body mass [25]. However, it is essential to note that we did not find a significant three-way interaction between the *FOLH1* 484T>C polymorphism, naturally occurring food folate intake, and sex. Therefore, no conclusions regarding sex-differences in the *FOLH1* by naturally occurring food folate intake interaction can be drawn from this work. Though our null finding may be due to a small sample size, our sex-stratified analyses suggest that future studies should perhaps consider possible sex effects.

The *FOLH1* 484T>C polymorphism has received little study in the literature, especially when compared to other well-known polymorphisms such as *FOLH1* 1561C>T. Specifically, the *FOLH1* 1561C>T polymorphism has been associated with both lower and higher RBC folate [54,55], serum folate, [4,54,55,56,57,58], and homocysteine levels [4,55,56,57,58,59], though some have failed to find such associations [60]. Furthermore, this polymorphism was not found to influence the bioavailability of monoglutamyl and polyglutamyl folic acid forms [54] and likewise has not been found to influence risk for NTDs [61]. In contrast, the *FOLH1* 484 T allele load was positively associated with RBC folate levels in a sample of schizophrenia patients, and was associated with treatment response to L-methylfolate and vitamin B12 supplementation [26]. Furthermore, this polymorphism has been associated with elevated homocysteine concentrations [58]. Though to our knowledge no previous study has evaluated the influence of *FOLH1* 484T>C polymorphism and dietary folate intake on RBC folate, our finding is nonetheless congruent with these previous results. While additional research is needed to clarify the functional role of the *FOLH1* 484T>C polymorphism, together these studies suggest a functional effect of the *FOLH1* 484 T>C SNP, such that the T-allele is associated with greater RBC folate levels, greater response of RBC folate to naturally occurring food folate intake, while the hypofunctional variant is associated with NTD risk. However, as this polymorphism remains poorly studied, additional research is needed to clarify the function of this SNP.

There are several important limitations to this study that must be taken into account. First, these results may have limited generalizability due to small sample size, single site design, and recruitment pool (i.e., individuals who were also willing to have an MRI scan). However, the validity of the current study is supported by the fact that the percentage of young women who fell below the EAR when considering folate intake from all sources (13.6%) was similar to that reported in a much larger epidemiologic study (15–19%) [22]. Furthermore, total folate intake from all sources for male (905 mcg DFE) and female (712 mcg DFE) participants was similar to that previously reported (813 mcg DFE and 724 mcg DFE respectively, [22]), and the distribution of *FOLH1* T484C alleles is consistent with that reported by larger sequencing studies (see https://www.ncbi.nlm.nih.gov/SNP/snp_ss.cgi?ss=ss1690360421). Despite the smaller size, this cohort is deeply phenotyped and presents the first interactive effects of the *FOLH1* 484T>C polymorphism and dietary intake on blood folate levels.

Second, nutritional intake was determined by self-report, which is subject to recall bias. A recent study that compared food frequency questionnaires to recovery biomarkers indicated substantial variation in the validity of intake self-report among nutrients [62]. It is unclear what specific factors would contribute to the over- or under-reporting of folate intake, but this remains a theoretical concern, and could potentially contribute to differences between reported folate intake in men and women. Though the use of quantitative estimates from such questionnaires has been previously reported in the literature [63,64,65], the definitive validation of such approaches for measuring long-term folate intake would require observation of the participant’s diet over a prolonged time not feasible for most studies [48]. Many suggest that such a comparison of quantitative nutrient estimates relative to dietary cutoffs is only an approximate unless individual variation is accounted for by modeling or in calibration studies [48,66]. Despite these issues, several DHQs have nonetheless been shown to provide reasonable estimates of nutrient intake [67,68,69,70]. While the DHQ used in this study has generally been found to be a valid assessment of nutrient intake as a whole [71], it has specifically been shown to show to provide a reasonable quantitative estimate of folate intake [72,73]. Nevertheless, though this measure may be a more reliable estimate of folate intake, it is critical to emphasize that the comparison of our findings with EAR values provides only qualitative evidence supporting the importance of fortification in maintaining dietary folate status. While interpreting our results relative to EAR cutoffs should be made with caution, as they only suggest the qualitative role of fortification in maintaining population folate status [48], that our results are consistent with those previously reported in larger studies affirms the importance of folic acid fortification intake for meeting population folate requirements [22].

Third, the strongest model emerging from our analysis still only accounted for less than 14 percent of the variance in RBC folate concentration (29% in female post-hoc analyses), indicating that the majority of variance in RBC folate levels reflects as-yet-to-be-determined factors. Though the significant interaction between naturally occurring food folate intake and the *FOLH1* 484T>C polymorphism explained only 2.3% of the variance in RBC folate levels, previous research has shown the individual contributions of SNPs to complex phenotypes such as RBC folate is generally low. For instance, Pvalíková and colleagues found that the well-studied *MTHFR* 677C>T variant specifically explained only 1% of the variance in RBC folate levels [74]. Importantly, recent research done in a sample unexposed to folic acid fortification found *FOLH1* 484 C homozygote mothers are at significantly greater risk of multiple-NTD-affected pregnancies compared to those with the functional variant [75]. Previous work suggests that the effect of certain polymorphisms on RBC folate status may be attenuated in high folate intake populations [53] while upregulation of *FOLH1* may be part of the physiological response to dietary folate deficiency [76]. Accordingly, the interaction of *FOLH1* 484T>C polymorphism and naturally occurring food folate may be of greater practical importance in populations for which RBC folate status is primarily determined by naturally occurring food folate, such as the high-NTD-risk population described by Guo and colleagues [75]. Therefore, additional studies are needed to better understand the effect of this polymorphism both on RBC folate levels, but also on risk for NTD-affected pregnancy, especially in populations that rely on naturally occurring food folate.

Finally, as RBC folate assays are not standardized, results obtained using our ECLIA could not be compared to epidemiological cutoffs for RBC folate insufficiency based on metabolic indicators or elevated risk of NTD-affected pregnancy established with other folate assays [31]. Furthermore, the lack of a standardized reference material for RBC folate measurement is problematic, as the accuracy of RBC measurements cannot be verified [49]. However, the use of a single assay platform with a coefficient of variation within the accepted range supports the validity of our RBC folate measurements for within study comparisons. Additionally, there is not yet consensus as to whether RBC folate is the preferable determinant of long-term folate status [for a review see 46]. However, RBC folate remains a sensitive indicator of long-term storage, reflecting folate status during erythropoiesis [77], is significantly correlated with liver folate stores [78], and has been shown to be a reasonable biomarker of NTD-risk [79]. Lastly, while previous research found different variants of the *MTHFR* 677C>T polymorphism are associated with altered distribution of folate forms within red blood cells [44], that the distribution of *MTHFR* alleles did not significantly differ between *FOLH1* T homozygotes and C-allele carriers suggests *FOLH1* results are likely not influenced by differential *MTHFR*-related folate recovery [45,46].

Despite its limitations, this study is consistent with previous research findings suggesting that while folic acid fortification and supplementation play a major role in boosting folate intake above EAR, many individuals still struggle to obtain sufficient folate as judged by this factor and as reported in previous work [22]. Of concern is the fact that 13.6 percent of women (whom were all of child-bearing age) were below the EAR for folate. The neural tube closes approximately four weeks after conception, which is earlier than the average time women recognize they are pregnant, 5.9 weeks [80]; this problem is compounded by the fact that approximately half of all pregnancies are unplanned [81]. Thus, it is crucial that all women capable of bearing children have sufficient folate intake regardless of whether they are actively trying to become pregnant, so as to ensure those who become pregnant have optimal periconceptional folate levels. Intake of synthetic folic acid may be particularly important in women who carry the *FOLH1* 484C allele, and who thus may have difficulty absorbing sufficient folate from natural sources.

As this variant has previously been associated with elevated risk of multiple-NTD-affected pregnancy [75], additional research is needed to further clarify both this association and any mechanisms by which *FOLH1* 484T>C contributes to NTD risk in different populations. Future research should therefore evaluate this *FOLH1* 484T>C polymorphism in both populations exposed and unexposed to mandatory folic acid fortification programs, to better clarify the practical significance of the interaction between naturally occurring food folate and the *FOLH1* 484 T>C polymorphism. As some have raised concerns about the tolerable upper limit of folate intake [82], future work should consider how individuals benefit from fortification if they are already taking a supplement with a high folic acid content. Lastly, the findings in this study support larger-scale investigation of determinants of blood folate, especially in women of childbearing age, as well as obstacles to achieving sufficient folate intake.

## 5. Conclusions

Among healthy young individuals exposed to folic acid fortification, blood folate level is dependent on relative intake from naturally occurring food folate and synthetic sources of folic acid, and on genetic variation in *FOLH1* 484 genotype. As predicted, naturally occurring food folate interacted with *FOLH1* 484 genotype, and only contributed to blood folate levels when this interaction was considered. In contrast, total synthetic folic acid intake contributed more directly to RBC levels, likely reflecting its lack of dependency on *FOLH1* for intestinal absorption. Finally, our analyses reinforce prior research regarding the importance of folic acid from fortified foods in maintaining adequate population folate status [22]. Additional research should clarify sex differences in absorption as well as the folate status of women of childbearing age both in the United States and in other countries with and without mandatory folic acid fortification of grain products.

## Figures and Tables

**Table 1:** Demographic characteristics of study participants.

	Total Sample (*n* = 265)	Males (*n* = 118)	Females (*n* = 147)	*p*-Value
Age (years) ± *SD*	23.6 ± 4.4	24.4 ± 4.5	22.9 ± 4.1	0.005
Self-reported race (White/non-White)	176/89	86/32	90/57	0.062
Education (years) ± *SD*	15.3 ± 2.5	15.4 ± 2.4	15.2 ± 2.6	0.46
*FOLH1* genotype (TT/C-carrier)	131/115	63/50	68/65	0.55

Demographic characteristics reported for the full sample and separately for males and females. Chi-square and independent samples *t*-tests were used to test the significance of differences between groups. *SD*, standard deviation; *FOLH1*; TT/C.

**Table 2:** Synthetic folic acid and naturally occurring food folate intake for male and female participants.

	Men	Women	*p*-Value
Variable	Mean ± *SD*	Mean ± *SD*	
Naturally occurring food folate (mcg)	361 ± 197	309 ± 211	0.0059
Folic acid from fortified foods (mcg DFE)	432 ± 285	266 ± 177	<0.0001
Supplemental folic acid (mcg DFE)	112 ± 221	136 ± 247	0.15
Folate from all sources (mcg DFE)	905 ± 482	712 ± 433	0.0002

Mean supplemental folic acid intake, folic acid intake from fortified foods, and naturally occurring food folate intake by sex. To facilitate comparison with naturally occurring food folate intake, intake of folic acid from fortified foods, supplemental folic acid, and folate from all sources are reported in mcg dietary folate equivalents (DFE).

**Table 3:** Sex differences in the percentage of participants below the Estimated Average Requirement (EAR).

	Percent of Men (*n* = 117) below EAR	Percent of Women (*n* = 147) below EAR	*p*-Value
Naturally occurring food folate	53.0	64.6	0.074
Food folate	6.0	20.4	0.0015
Folate from all sources	6.0	13.6	0.068

Percentage of male and female participants who report folate intake below the EAR when considering naturally occurring food folate intake, food folate, and folate intake from all sources.

**Table 4:** Predictors of red blood cell (RBC) folate via hierarchical linear regression (*n* = 180).

Model Number	Variables	Beta	*p*-Value	Adjusted R^2^	R^2^ Change	Significance of R^2^ Change
1	Log of naturally occurring food folate	0.07	0.37	−0.001	0.	0.37
2	Log of naturally occurring food folate	−0.09	0.26	0.0725	0.0736	<0.001
	Log of total synthetic folic acid	0.32	<0.001			
3	Log of naturally occurring food folate	−0.08	0.31	0.0836	0.0111	0.078
	Log of total synthetic folic acid	0.32	<0.001			
	Genotype	0.13	0.078			
4	Log of naturally occurring food folate	−0.13	0.27	0.0801	−0.0036	0.57
	Log of total synthetic folic acid	0.32	<0.001			
	Genotype	−0.27	0.70			
	Genotype x log of naturally occurring food folate interaction	0.40	0.57			

The log of naturally occurring food folate and total synthetic folic acid were used since the data for these categories were not normally distributed (Shapiro–Wilk *p*’s < 0.001). For *FOLH1* genotype, participants were characterized as either a C carrier (C/C or C/T) or T/T due to the low frequency (*n* = 11) of the homozygous CC genotype.

**Table 5:** Caucasian stratified predictors of RBC folate via hierarchical linear regression (*n* = 123).

Model Number	Variables	Beta	*p*-Value	Adjusted R^2^	R^2^ Change	Significance of R^2^ Change
1	Log of naturally occurring food folate	0.03	0.77	−0.008	0.	0.77
2	Log of naturally occurring food folate	−0.17	0.082	0.114	0.121	<0.001
	Log of total synthetic folic acid	0.41	<0.001			
3	Log of naturally occurring food folate	−0.17	0.079	0.115	0.001	0.27
	Log of total synthetic folic acid	0.41	<0.001			
	Genotype	0.09	0.2773			
4	Log of naturally occurring food folate	−0.42	0.008	0.138	0.023	0.043
	Log of total synthetic folic acid	0.43	<0.001			
	Genotype	−10.87	0.054			
	Genotype x log of naturally occurring food folate interaction	10.99	0.043			

The log of naturally occurring food folate and total synthetic folic acid were used since the data for these categories were not normally distributed in the full sample (Shapiro–Wilk *p*’s < 0.001). For *FOLH1* genotype, participants were characterized as either a C carrier (C/C or C/T) or T/T due to the low frequency of the homozygous CC genotype.

**Table 6:** Predictors of RBC folate in Caucasian females and males via hierarchical linear regression.

Model Number	Variables	Beta	*p*-Value	Adjusted R^2^	R^2^ Change	Significance of R^2^ Change
1	Log of naturally occurring food folate	−0.15 (0.18)	0.27 (0.14)	0.004 (0.018)	0.	0.27 (0.14)
2	Log of naturally occurring food folate	−0.32 (−0.003)	0.014 (0.98)	0.191 (0.086)	0.187 (0.068)	<0.001 (0.021)
	Log of total synthetic folic acid	0.48 (0.34)	<0.001 (0.021)			
3	Log of naturally occurring food folate	−0.32 (−0.01)	0.016 (0.94)	0.178 (0.091)	−0.0139 (0.005)	0.79 (0.25)
	Log of total synthetic folic acid	0.48 (0.35)	<0.001 (0.018)			
	Genotype	0.03 (0.14)	0.79 (0.25)			
4	Log of naturally occurring food folate	−0.88 (−0.07)	<0.001 (0.72)	0.29 (0.071)	0.112 (−0.02)	0.003 (0.67)
	Log of total synthetic folic acid	0.54 (0.35)	<0.001 (0.019)			
	Genotype	−40.17 (−0.44)	0.0036 (0.75)			
	Genotype x log of naturally occurring food folate interaction	40.25 (0.59)	0.0033 (0.67)			

Results for male participants (*n* = 65) are presented in parentheses below those for female participants (*n* = 58). The log of naturally occurring food folate and total synthetic folic acid were used since the data for these categories were not normally distributed in the full sample (Shapiro–Wilk *p*’s < 0.001). For *FOLH1* genotype, participants were characterized as either a C carrier (C/C or C/T) or T/T due to the low frequency of the homozygous CC genotype.

## Supplementary Materials

The following are available online at www.mdpi.com/2072-6643/9/9/994/s1, Table S1: List of folate terms, Table S2: Demographics of participants by recruitment source, Table S3: Demographic differences of participants included and excluded in the regression, Table S4: Synthetic folic acid predictors of RBC folate status.
