# Supplementary material for: A Cross-Sectional Study of Dietary and Genetic Predictors of Blood Folate Levels in Healthy Young Adults
Source: Nutrients. 2017 Sep 8;9(9):994. doi: 10.3390/nu9090994 (PMC5622754; doi:10.3390/nu9090994)
Supplement: Supplementary file 1 [file nutrients-09-00994-s001.pdf]

Table S1. List of folate terms

Term	Definition
**Naturally Occurring Food Folate**	Polyglutamate folate form found naturally in foods and not added through fortification
**Folic Acid from Fortified Foods**	Synthetic folic acid added to certain foods through a process known as fortification
**Food Folate**	Total folate derived from food, reflecting both folic acid from fortified foods and naturally occurring food folate
**Supplemental Folic Acid**	Synthetic folic acid intake from vitamins
**Folate from All Sources**	Total folate derived from naturally occurring food folate, folic acid from fortified foods, and supplemental folic acid
**Total Synthetic Folic Acid**	Total synthetic acid from supplements (supplemental folic acid) and folic acid from fortified foods

Folate terms used to describe different sources of folate with brief definitions.

Table S2. Demographics of participants by recruitment source

	**Facebook (*n*=33)**	**Craigslist (*n*=159)**	**Hospital (*n*=73)**
**Age (years) ± *SD***	18.5 ± 0.6	24.0 ± 4.4	24.9 ± 3.7
**Self-reported Race (White / non-White)**	18 / 15	106 / 53	52 / 21
**Education (years) ± *SD***	12.2 ± 1.3	15.4 ± 2.4	16.5 ± 2.0
**Sex (Male / Female)**	11 / 22	81 / 78	26 / 47

Demographic information reported for participants recruited from Facebook, Craigslist, and from Hospital-wide electronic recruitment resources.

Table S3. Demographic differences of participants included and excluded in the regression

	**Included (*n* = 180)**	**Excluded (*n* = 85)**	**Statistics**	***p*-value**
**Age (years) ± *SD***	23.9 ± 4.5	22.8 ± 4.0	$t = 1.97$	0.06
**Self-reported Race (White / non-White)**	123 / 57	53 / 32	$\chi^2 = .68$	0.41
**Education (years) ± *SD***	15.4 ± 2.5	15.1 ± 2.5	$t = 1.06$	0.29
***FOLH1* genotype (TT / C-carrier)**	94 / 86	37 / 29	$\chi^2 = 0.15$	0.70
**Sex (Male / Female)**	85 / 95	52 / 33	$\chi^2 = 1.33$	0.25

Demographic characteristics reported for participants included in and excluded from the regression analysis. Chi-square and independent samples *t*-tests were used to test the significance of differences between groups.

Table S4. Synthetic folic acid predictors of RBC folate status

Variables	Beta	p-value	Adjusted R^2	Significance of R^2 Change
**Log of supplemental folic acid**	0.20	0.006	0.059	0.002
**Log of folic acid from fortified foods**	0.17	0.02		

Regression of log-transformed synthetic folic acid intake (from supplements and from fortified foods) on RBC folate level.

MTHFR **Supplemental Analysis**

Prior research suggests different variants of the common *MTHFR* C677T polymorphism are associated with an altered distribution of folate forms in red blood cells [44], which could in turn influence RBC folate measurements [45,46]. Therefore, we conducted a one-way ANOVA to determine whether RBC folate levels differed amongst the three *MTHFR* genotypes. Additionally we then used chi-square tests to determine whether the frequency of *MTHFR* genotypes differed between the two *FOLH1* groups (T/T and C-carrier) for all subjects with valid genotype, nutrition, and RBC folate data.

For all Caucasian subjects with valid genotype and RBC folate measurements included in the later regression analysis (n = 123) there were no significant differences in RBC folate level across each of the three *MTHFR* genotypes (CC, 36.6%; CT, 49.6%; and TT, 13.8%), $F(2,120) = 1.62$, $p = 0.20$. As previous research suggests *MTHFR* effects on RBC folate may be primarily driven by the T allele [53], we conducted a chi-square test to see if the distribution of *MTHFR* genotypes differed between *FOLH1* 484 T-allele homozygotes and C-allele carriers. There was no significant difference in the frequency of *MTHFR* genotypes between the *FOLH1* T-allele homozygotes (CC, 40.7%; CT, 44.4%; TT, 14.8%) and C-allele carriers (CC, 33.3%; CT, 53.6%; TT, 13.0%), $\chi^2 = 1.04$, $p = 0.60$. Consequently, *MTHFR* genotype was not accounted for in regression modeling.
